# 1395. COVID 19 Incidence rates were not different among high risk health-care workers and non-health workers after SARS-CoV-2 vaccination: a prospective study in Cali-Colombia.

**DOI:** 10.1093/ofid/ofac492.1224

**Published:** 2022-12-15

**Authors:** Fernando Rosso, Laura A Torres-Canchala, Isabel L Zapata-Vasquez, Julio Llanos-Torres, Eric Tafur, Jenny P Muñoz-Lombo, Ludwig Albornoz, Sergio I Prada-rios

**Affiliations:** Fundación Valle del Lili, Cali, Valle del Cauca, Colombia; Fundacion Valle del Lili, Cali, Valle del Cauca, Colombia; Fundacion Valle del Lili, Cali, Valle del Cauca, Colombia; Fundacion Valle del Lili, Cali, Valle del Cauca, Colombia; Universidad Icesi, Cali, Valle del Cauca, Colombia; Universidad Icesi, Cali, Valle del Cauca, Colombia; Fundacion Valle del Lili, Cali, Valle del Cauca, Colombia; Fundacion Valle del Lili, Cali, Valle del Cauca, Colombia

## Abstract

**Background:**

The seroprevalence of COVID-19 among health-care workers (HCWs) is still not well characterized in Latin America and the Caribbean. The objective of this study was to compare incidence rates (IR) during the COVID-19 pandemic among HCWs vs. non-HCWs in a university hospital in Cali, Colombia.

**Figure 1:**
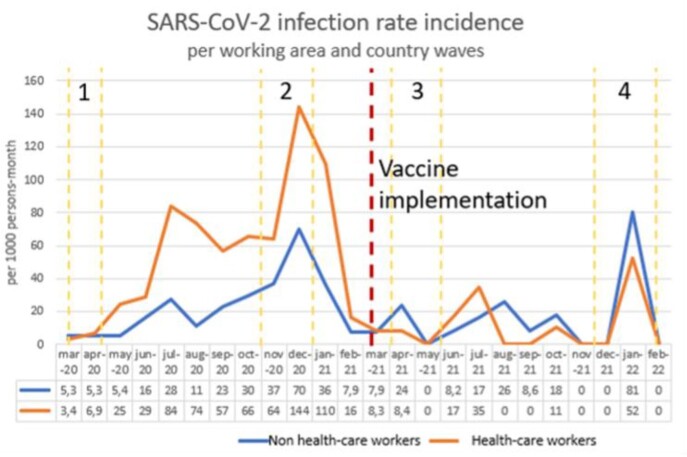

**Figure 2:**
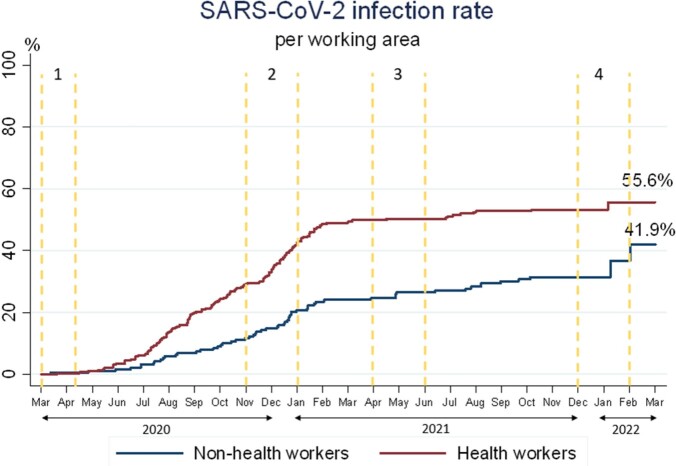

**Methods:**

A prospective study was performed. The study included two groups: HCWs with high-risk contact of SARS-CoV-2 infections vs. administrative hospital workers (non-HCW). Seroprevalence of SARS-CoV-2 antibodies between both groups was compared according to vaccination history and confirmed SARS-CoV-2 infection during follow-up (March 6^th^, 2020, to February 28^th^, 2022). The study was developed in three phases according to the infection waves in Colombia, measuring antibodies anti-nucleocapsid and anti-spike serum concentration in each one. A descriptive analysis was done to compare both groups and IR per month (Figure 1).

**Results:**

480 participants were included, 291 (60.6%) were HCWs, and 189 (39.4%) were non-HCWs. After the second wave and before vaccination, the accumulative seroprevalence was 40.6%: 49.1% of HCWs vs. 27.5% of non-HCWs (p< 0.001). 9.2% of HCWs and 7.9% of non-HCWs seropositive individuals had an asymptomatic infection (p=0.447). Of the 51.9% of susceptible HCWs and 72.5% of susceptible non-HCWs before the third wave, the risk of developing SARS-CoV-2 infection was 9.2% and 12.8%, respectively. After 24 months, the infection rate was higher in HCWs and non-HCWs (55.6% vs. 41.9%, p< 0.001) (Figure 2). The total IR was 31.4/1,000 person-month, with an IR difference of 21/1,000 person-month being higher in HCWs comparing non-HCWs (40.7 vs. 19.8, p< 0.001), but after vaccination (April 2021), the IR difference was not significative (IR difference 5%, p=0.1605). The asymptomatic disease was 9.8% of HCWs vs. 10.2% of non-HCWs. Since vaccination, 93.6% of workers had positive anti-S antibodies after 2 doses; and 100% had them after 3^rd^ dose. SARS-CoV-2 Omicron variant increased cases during the fourth wave, more in non-HCWs.

**Conclusion:**

Before vaccination, HCWs had higher infection rates, mainly after the second wave. However, after the immunization, the IR in both groups significantly decreased and equalized in both groups.

**Disclosures:**

**All Authors**: No reported disclosures.

